# Quantum computational speed of a nanowires system with Rashba interaction in the presence of a magnetic field

**DOI:** 10.1038/s41598-021-02051-2

**Published:** 2021-11-23

**Authors:** Rabie I. Mohamed, Manal G. Eldin, Ahmed Farouk, A. A. Ramadan, M. Abdel-Aty

**Affiliations:** 1 Mathematics and Computer Science Department, Faculty of Science, Beni-Suef University, Beni-Suef, Egypt; 2 Department of Computer Science, Faculty of Computers and Artificial Intelligence, South Valley University, Hurghada, Egypt; 3 Department of Mathematics, Faculty of Science, Sohag University, Sohâg, 82524 Egypt

**Keywords:** Information theory and computation, Optical physics, Quantum physics, Nanowires, Applied mathematics

## Abstract

The present research is designed to examine the dynamic of the quantum computational speed in a nanowire system through the orthogonality speed when three distinct types of magnetic fields are applied: the strong magnetic field, the weak magnetic field, and no magnetic field. Moreover, we investigate the action of the magnetic fields, the spin-orbit coupling, and the system’s initial states on the orthogonality speed. The observed results reveal that a substantial correlation between the intensity of the spin-orbit coupling and the dynamics of the orthogonality speed, where the orthogonality speed decreasing as the spin-orbit coupling increases. Furthermore, the initial states of the nanowire system are critical for regulating the speed of transmuting the information and computations.

## Introduction

The quantum computational speed has a vital role in quantum communication^1^, quantum information processing^2, 3^, and quantum computation^4^. The orthogonality speed refers to the shortest time necessary for transition the quantum system from one orthogonal state (node) to another, and it is used to detect the speed of computations^2, 5^. Many research groups have studied the orthogonality speed for a single or two-qubit state. For a single qubit system interacting with a quantized field or interacting with a rectangular pulse, the speed of orthogonality has been explored^6, 7^, while it was investigated in a two-qubit system interacting with various types of spin interaction^8^. To our knowledge, no published studies on the effect of applied magnetic and electric fields on quantum computational speed in a nanowire system. As a result, in the current work, we study the pattern of the orthogonality speed in a novel system consisting of a ballistic nanowire excited by Rashba spin-orbit coupling (RSOC) in the presence or absence the perpendicular magnetic fields when system’s initial states are prepared in several forms: pure state, maximum entangled state, and superposition state. We find that the magnetic field, the spin-orbit coupling strength and the system’s initial states can be used to control the computational speed of a nanowire system.

Nowadays with the development of semiconductor manufacture technology, we have reached low-dimensional systems with short length scales such as quantum well, quantum wire, and quantum dot where the motion and dynamics of charge carriers (electrons) can only be interpreted via the law of quantum mechanics^9, 10^. The study of physical characteristics such as transport, optic, and electrical properties of low dimensional semiconductor structures has sparked a lot of interest due to their potential application in the manufacture of a wide range of microelectronic devices, optoelectronic devices, and fluorescent devices^11, 12^.

The effect of magnetic and electric fields on the transport properties, optical characteristics, and electronic structure of low-dimensional semiconductor systems has been investigated in numerous research^13–25^. For example, the effect of an in-plane electric field on a two-dimensional electron system with RSOC in the presence of a magnetic field has been investigated^13^. A perpendicular magnetic field’s effects on the spin and spectral characteristics of a ballistic quantum well system with the Rashba impact have been studied^14^. Also, the influence of an external electric field on the optical absorption of a nanowire exposed to a perpendicular magnetic field and the Rashba effect has been estimated^15^. The impacts of the magnetic field, RSOC, and external electric field on a quantum wire to determine the dispersion relations and effective g-factor for different spin split subbands have been examined^16^. Moreover, The influence of the SOC and the magnetic field on the energy levels of a quasi-one-dimensional quantum wire have been investigated^17^. The thermodynamic properties of a nanowire under the Rashba spin-orbit interaction and external magnetic field have been studied^24^.

On the other hand, the effects of the SOC and the magnetic field intensities on the nanowire systems have received less attention in quantum information and quantum computing. Exceptionally in the last years for quantum information, in the presence of strong and weak magnetic fields the dynamical behaviour of squeezing in a nanowire system with Rashba interaction has been explored^26^. Furthermore, the quantum correlations in a ballistic quantum wire under RSOC within a magnetic field have been studied^27^. The energy-level crossing and the quantum Fisher information behavior in a two-dimensional quantum wire interacting with Rashba and Dresselhaus SOC have been explored^28^. In addition, when the weak and strong magnetic fields are employed, the quantum entanglement of a nanowire system through negativity has been discussed^29^. While, in quantum computing a new application of a ballistic nanowire system with SOC to create specific new quantum gates has been investigated^30^.

This paper is organized as follows: In “The physical model” section, we offer an analytical solution to the physical model when three distinct types of magnetic fields are employed to the nanowire system with RSOC. In “The orthogonality speed” section, we use the orthogonality speed to estimate the quantum computational speed when the system’s initial states are prepared in three different forms. The effects of the magnetic field, the SOC, and the system’s initial state on the computational speed of a nanowire system are discussed in “Results and discussion” section. Our conclusion will be presented in the last section.

## The physical model

Let us consider a semiconductor quantum wire with a parabolic confinement potential in the x-direction given as $V(x)=\frac{1}{2} m \omega ^{2} x^{2}$ characterized by the harmonic oscillator frequency $\omega$ and effective mass *m*. The Rashba effect occurs in this system due to the asymmetry of the structure, which is defined by $H_{r} = \frac{\alpha _{r}}{\hbar }\left[ (p_{y}+e B x){\hat{\sigma }}_{x} -p_{x} {\hat{\sigma }}_{y}\right]$ with the strength of Rashba interaction $\alpha _{r}$. Therefore, the total Hamiltonian of this system when the perpendicular magnetic field $\overrightarrow{B} = B {\widehat{e}}_z$ is employed in the positive z-direction can be expressed as^14, 15, 26, 31^:

1 $$\begin{aligned} H=\frac{\left[ p_{x}^{2}+(p_{y}+e B x)^{2}\right] }{2m}+ \frac{1}{2} m \omega ^{2} x^{2} + e F x + \frac{\textit{g} }{2}\mu _B B {\hat{\sigma }}_{z} + \frac{\alpha _{r}}{\hbar }\left[ (p_{y}+e B x){\hat{\sigma }}_{x} -p_{x} {\hat{\sigma }}_{y}\right] , \end{aligned}$$

Here, *F* is the external electric field, $\overrightarrow{p}=(p_{x},p_{y})$ is the linear momentum, *g* is Lande’s *g*-factor, *e* is the electronic charge, $\overrightarrow{\sigma }=(\sigma _{x}, \sigma _{y}, \sigma _{z})$ are the Pauli matrices, and $\mu _B$ is the Bohr magneton.

In units of $\hbar \omega$ the Hamiltonian (1) can be expressed in the following dimensionless form by the ladder operators of a shifted harmonic oscillator ${\hat{a}}^\dag$ and ${\hat{a}}$ as:

2 $$\begin{aligned} H=\Omega \left( {\hat{a}}^\dag {\hat{a}}+\frac{1}{2}\right) +\frac{1}{2}(\eta +\xi _1 {\hat{\sigma }}_x+\delta {\hat{\sigma }}_z +\xi _2({\hat{a}}^\dag +{\hat{a}}){\hat{\sigma }}_x+ i \xi _3({\hat{a}}-{\hat{a}}^\dag ){\hat{\sigma }}_y ). \end{aligned}$$

In Eq. (2) we used the typical length scales $l_o=\sqrt{\frac{\hbar }{m \omega }}$, $l_B=\sqrt{\frac{\hbar }{m \omega _c}}$, and $l_{so}=\frac{\hbar ^2}{2 m \alpha _{r}}$ to describe the strengths of the confinement potential, the magnetic field, and RSOC respectively with the oscillator frequency $\omega _c =\frac{eB}{m}$. So, the dimensionless Zeeman splitting is $\delta =\frac{\textit{g}}{2} \frac{m}{m_o} \left( \frac{l_o}{l_B} \right) ^2$ with the free electron mass $m_o$. Also, the remaining different quantities above are defined as follows:

$$\begin{aligned} \Omega&=\sqrt{1+\left( \frac{l_o}{l_B}\right) ^4}, \; \eta =(l_o k)^2-\left( \frac{ \Omega \chi _c }{l_o}\right) ^2, \; \chi _c =\frac{l_o}{\Omega ^2}\left[ l_o K_F +l_o k \left( \frac{l_o }{l_B}\right) ^2\right] , \; K_F = \frac{e F}{\hbar \omega },\\ \xi _1&=\frac{l_o}{l_{so}}\left( l_o k-\frac{l_o \chi _c}{l_B^2}\right) , \; \xi _2=\frac{1}{\sqrt{2\Omega }}\frac{l_o}{l_{so}}\left( \frac{l_o}{l_B}\right) ^2, \; and \;\;\; \xi _3=\sqrt{\frac{\Omega }{2}} \frac{l_o}{l_{so}} \end{aligned}$$

Here, we study the Hamiltonian of a nanowire system when a three different type of magnetic field are applied.

(i) *When the magnetic field is weak* $l_B \gg l_o$
  The Hamiltonian in Eq. (2) when the weak magnetic field $l_B \gg l_o$ is applied, under condition the external electric field $F= k \hbar \omega \left( \frac{\Omega ^{2}\omega }{e \omega _{c}}-\frac{\omega _{c}}{e\omega } \right)$, and $l_o K_F = l_o k \ll 1$, with a rotating-wave approximation(RWA) written as:
  3 $$\begin{aligned} H_{1}=\Omega \left( {\hat{a}}^\dag {\hat{a}}+\frac{1}{2}\right) +\frac{\delta }{2} {\hat{\sigma }}_z +\left( \frac{\xi _2+\xi _3}{2}\right) ({\hat{a}}{\hat{\sigma }}_{+}+{\hat{a}}^\dag {\hat{\sigma }}_{-}), \end{aligned}$$
  where, $${\hat{\sigma }}_{\pm }={\hat{\sigma }}_{x}\pm i{\hat{\sigma }}_{y}$$.
  The time evolution of Hamiltonian (3) is governed by the Schrödinger equation $i\frac{\partial |\Psi (t)\rangle }{\partial t} = H_{1} |\Psi (t)\rangle$ with $\hbar =1$, and the wave function of the two lowest levels energy is $|\Psi (t)\rangle =\alpha _{1}(t)|1\rangle +\alpha _{2}(t)|2\rangle +\alpha _{3}(t)|3\rangle + \alpha _{4}(t)|4\rangle$ with the space harmonic-electron states $\{|1\rangle =|\text {g}, 0\rangle , |2\rangle =|\text {g}, 1\rangle , |3\rangle =|\text {e}, 0\rangle , |4\rangle =|\text {e}, 1\rangle \}$ where the states $|0\rangle$ and $|1\rangle$ correspond to the states of the harmonic oscillator, while the states $|e\rangle$ and $|g\rangle$ represent the excited and ground state of the electron spin. Therefore, the solution of this equation is given by $|\Psi (t)\rangle = {\widehat{U}} |\Psi (0)\rangle$, where the unitary operator ${\widehat{U}}$ given by:
  4 $$\begin{aligned} {\widehat{U}}=e^{-i\Omega t}\left[ e^{i P_{1}t}|1\rangle \langle 1|+\mu _{-}|2\rangle \langle 2|+\mu _{+}|3\rangle \langle 3| -i \frac{\lambda }{u}\sin (u t)(|2\rangle \langle 3|+|3\rangle \langle 2|)+e^{-i P_{1}t}|4\rangle \langle 4|\right] , \end{aligned}$$
  where,
  5 $$\begin{aligned} \begin{aligned} \mu _{\pm }&=\left( \cos (u t)\pm i \frac{P_{2} }{u}\sin (u t) \right) ,\quad P_{1}=\frac{(\Omega +\delta )}{2},\\ u&=\sqrt{(P_{2})^{2}+(\lambda )^{2}}, \qquad P_{2}=\frac{(\Omega -\delta )}{2}, \qquad \lambda =\frac{(\xi _2+\xi _3)}{2} \end{aligned} \end{aligned}$$
(ii) *When the magnetic field is strong* $l_B \ll l_o$
  With the same conditions in (i) the Hamiltonian (2) if the robust magnetic field $l_B \ll l_o$ is employed, can be written as:
  6 $$\begin{aligned} H_{2}=\left( {\hat{a}}^\dag {\hat{a}}+\frac{1}{2}\right) +\frac{\textit{g}}{4}\frac{m}{m_o} {\hat{\sigma }}_z +\frac{1}{\sqrt{2}}\frac{l_B}{l_{so}}({\hat{a}}{\hat{\sigma }}_{+}+{\hat{a}}^\dag {\hat{\sigma }}_{-}), \end{aligned}$$
  the analytical solution of the Hamiltonian system (6) is similar to the solution in the case of the weak magnetic field in Eq. (4) with minor differences in the following quantities:
  7 $$\begin{aligned} \Omega =1, \qquad \delta =\frac{1}{2}\frac{m}{m_o}\textit{g}, \qquad \lambda =\frac{1}{\sqrt{2}}\frac{l_B}{l_{so}} \end{aligned}$$
(iii) * In the absence of magnetic field* $B = 0$
  Finally, the Hamiltonian (2) of a nanowire system when no magnetic field is employed $B = 0$ is given by,
  8 $$\begin{aligned} H_{3}=\left( {\hat{a}}^\dag {\hat{a}}+\frac{1}{2}\right) +\frac{1}{\sqrt{8}}\frac{l_o}{l_{so}}({\hat{a}}{\hat{\sigma }}_{+}+{\hat{a}}^\dag {\hat{\sigma }}_{-}), \end{aligned}$$

also, the solution of the Hamiltonian (8) is similar to the unitary operator (4), but with different parameters as:

9 $$\begin{aligned} \Omega =1, \qquad \delta =0, \qquad \lambda =\frac{1}{\sqrt{8}}\frac{l_o}{l_{so}} \end{aligned}$$

## The orthogonality speed

In this section, we use the orthogonality to explore the quantum computational speed when the nanowire system is constructed in three distinct starting states as: the pure state, the maximum entangled state, and the superposition state. Let us consider that, the proposed system is prepared initially in the state $|\Psi (0)\rangle$ which evolved for a time t with the final state $|\Psi (t)\rangle = {\widehat{U}} |\Psi (0)\rangle$. Therefore, the orthogonality can be defined by the scalar product of the vectors as the following^3, 6, 7^:

10 $$\begin{aligned} S_{or}= \langle \Psi (0)|\Psi (t)\rangle \end{aligned}$$

### When the system’s initial state in the pure state

Assume that the nanowire model is constructed initially in the pure state $|\Psi (0)\rangle = |e, 0\rangle$. The eigenvectors of this state is obtained as,

11 $$\begin{aligned} \phi _{1}(0) = \{1, 0, 0, 0\}, \phi _{2}(0) = \{0, 1, 0, 0\}, \phi _{3}(0) = \{0, 0, -1, 0\}, \phi _{4}(0) = \{0, 0, 0, 1\} \end{aligned}$$

Then, one can compute the time evolution of the density operator for this initial pure state as the following:

12 $$\begin{aligned} {\hat{\rho }}_{AB}^{P}(t) =\rho _{33} |3\rangle \langle 3|+\rho _{32} |3\rangle \langle 2|+ \rho _{23} |2\rangle \langle 3|+\rho _{22} |2\rangle \langle 2|, \end{aligned}$$

where, the density operator elements are given by,

13 $$\begin{aligned} \rho _{33}= \mid \mu _{+} \mid ^{2},\qquad \rho _{32}= i \frac{\lambda \mu _{+}}{u} \sin (u t),\qquad \rho _{22}= \frac{\lambda ^{2}}{u^{2}} \sin ^{2}(u t), \qquad \rho _{23}=\rho ^{*}_{32} \end{aligned}$$

Therefore, the eigenvectors of the final state ${\hat{\rho }}_{AB}^{P}(t)$ can be written as follows:

14 $$\begin{aligned} \begin{aligned} \psi _{1}(t)&= \{1, 0, 0, 0\},\quad \psi _{2}(t) = \{0, 0, 0, 1\},\\ \psi _{3}(t)&=\left\{ 0, \frac{ | \rho _{32}|\gamma _{-}}{\rho _{32} \sqrt{|\rho _{32}|^{2}+| \gamma _{-}|^{2}}}, \frac{ | \rho _{32}|}{ \sqrt{|\rho _{32}|^{2}+| \gamma _{-}|^{2}}}, 0 \right\} , \\ \psi _{4}(t)&= \left\{ 0, \frac{ | \rho _{32}|\gamma _{+}}{\rho _{32} \sqrt{|\rho _{32}|^{2}+| \gamma _{+}|^{2}}}, \frac{ | \rho _{32}|}{ \sqrt{|\rho _{32}|^{2}+| \gamma _{+}|^{2}}}, 0\right\} , \end{aligned} \end{aligned}$$

where, $$\gamma _{\pm }=\frac{1}{2}\left[ (\rho _{22}-\rho _{33})\pm \sqrt{(\rho _{22}-\rho _{33})^{2}+4|\rho _{32}|^{2}}\right]$$

### When the system’s initial state in the maximum entangled state

Also, if the system is initially created in the maximum entangled state $|\Psi (0)\rangle = \frac{1}{\sqrt{2}}(|\text {e}, 0\rangle +|\text {g}, 1\rangle )$, the eigenvectors of this state become:

15 $$\begin{aligned} \phi _{1}(0) = \{1, 0, 0, 0\}, \phi _{2}(0) = \{0, 0, 0, 1\}, \phi _{3}(0) = \left\{ 0, \frac{1}{\sqrt{2}}, \frac{1}{\sqrt{2}}, 0\right\} , \phi _{4}(0) = \left\{ 0, - \frac{1}{\sqrt{2}}, \frac{1}{\sqrt{2}}, 0\right\} . \end{aligned}$$

Moreover, the time evolution of the final state for this initial maximum entangled state can be calculated as:

16 $$\begin{aligned} {\hat{\rho }}_{AB}^{M}(t) =\frac{1}{2}\left( \mid r_{1} \mid ^{2}|2\rangle \langle 2|+ r_{1} r_{2}^{*} |2\rangle \langle 3|+r_{2} r_{1}^{*} |3\rangle \langle 2|+\mid r_{2} \mid ^{2} |3\rangle \langle 3| \right) , \end{aligned}$$

where,

17 $$\begin{aligned} r_{1}=\mu _{-}-i \frac{\lambda }{u} \sin (u t), \qquad r_{2}=\mu _{+}-i \frac{\lambda }{u} \sin (u t). \end{aligned}$$

Then, the eigenvectors of the final state Eq. (16) are the same eigenvectors in Eq. (14), but the density operator elements are different which given by,

18 $$\begin{aligned} \rho _{22}=\frac{1}{2} \mid r_{1} \mid ^{2},\qquad \rho _{23}= \frac{1}{2} ( r_{1} r_{2}^{*} ) ,\qquad \rho _{33}= \frac{1}{2} \mid r_{2} \mid ^{2}, \qquad \rho _{32}=\rho ^{*}_{23} \end{aligned}$$

### When the system’s initial state in the superposition state

Finally, we consider the nanowire model is set up in the superposition state $|\Psi (0)\rangle = \frac{1}{2}(|\text {g}, 0\rangle +\text {e}, 0\rangle + |\text {g}, 1\rangle |+ \text {e}, 1\rangle )$. The eigenvectors corresponding to this state can be calculated as:

19 $$\begin{aligned} \begin{aligned} \phi _{1}(0)&= \left\{ \frac{1}{2}, \frac{1}{2}, \frac{1}{2}, \frac{1}{2}\right\} , \quad \quad \quad \phi _{2}(0) = \left\{ -\frac{1}{\sqrt{2}}, 0, 0, \frac{1}{\sqrt{2}}\right\} ,\\ \phi _{3}(0)&=\left\{ - \frac{1}{\sqrt{2}}, 0, \frac{1}{\sqrt{2}}, 0\right\} , \quad \phi _{4}(0) = \left\{ - \frac{1}{\sqrt{2}}, \frac{1}{\sqrt{2}}, 0, 0\right\} . \end{aligned} \end{aligned}$$

The density operator for this initial superposition state is given by:

20 $$\begin{aligned} {\hat{\rho }}_{AB}^{S}(t)=\sum _{i,j=1}^{4} \rho _{i j} |i\rangle \langle j| \end{aligned}$$

If the electron’s spin states $(|\text {g}\rangle , |\text {e}\rangle )$ are described by subsystem *A*, and the harmonic oscillator’s orbital states $(|0\rangle , |1\rangle )$ are represented by subsystem *B*, then the reduced density matrix for subsystem *B* is as follows:

21 $$\begin{aligned} {\hat{\rho }}_{B}^{S}(t) =\rho _{11} |1\rangle \langle 1|+ \rho _{12} |1\rangle \langle 2|+\rho _{21} |2\rangle \langle 1|+\rho _{22} |2\rangle \langle 2| +\rho _{33} |3\rangle \langle 3|+\rho _{34} |3\rangle \langle 4|+\rho _{43} |4\rangle \langle 3|+\rho _{44} |4\rangle \langle 4|, \end{aligned}$$

where, the reduced density matrix elements ${\hat{\rho }}_{B}^{S}(t)$ are ,

22 $$\begin{aligned} \begin{aligned} \rho _{11}&= \rho _{44}=\frac{1}{4}, \qquad \rho _{22}=\frac{1}{4} \mid r_{1} \mid ^{2}, \qquad \rho _{33}= \frac{1}{4} \mid r_{2} \mid ^{2},\\ \rho _{12}&= \frac{1}{4} ( r_{1}^{*} e^{i P_{1}t}), \qquad \rho _{21}=\rho ^{*}_{12}, \qquad \rho _{34}= \frac{1}{4} ( r_{2} e^{i P_{1}t}), \qquad \rho _{43}=\rho ^{*}_{34}. \end{aligned} \end{aligned}$$

The eigenvectors of the final state ${\hat{\rho }}_{B}^{S}(t)$ are given by:

23 $$\begin{aligned} \begin{aligned} \psi _{1}(t)&=\left\{ \frac{ | \rho _{21}|\alpha _{-}}{\rho _{21} \sqrt{|\rho _{21}|^{2}+| \alpha _{-}|^{2}}}, \frac{ | \rho _{21}|}{ \sqrt{|\rho _{21}|^{2}+| \alpha _{-}|^{2}}}, 0, 0\right\} , \\ \psi _{2}(t)&=\left\{ \frac{ | \rho _{21}|\alpha _{+}}{\rho _{21} \sqrt{|\rho _{21}|^{2}+| \alpha _{+}|^{2}}}, \frac{ | \rho _{21}|}{ \sqrt{|\rho _{21}|^{2}+| \alpha _{+}|^{2}}}, 0, 0\right\} , \\ \psi _{3}(t)&=\left\{ 0, 0, \frac{ | \rho _{43}|\beta _{-}}{\rho _{43} \sqrt{|\rho _{43}|^{2}+| \beta _{-}|^{2}}}, \frac{ | \rho _{43}|}{ \sqrt{|\rho _{43}|^{2}+| \beta _{-}|^{2}}} \right\} , \\ \psi _{4}(t)&= \left\{ 0, 0, \frac{ | \rho _{43}|\beta _{+}}{\rho _{43} \sqrt{|\rho _{43}|^{2}+| \beta _{+}|^{2}}}, \frac{ | \rho _{43}|}{ \sqrt{|\rho _{43}|^{2}+| \beta _{+}|^{2}}} \right\} , \end{aligned} \end{aligned}$$

where,

24 $$\begin{aligned} \alpha _{\pm }=\frac{1}{2}\left[ (\rho _{11}-\rho _{22})\pm \sqrt{(\rho _{11}-\rho _{22})^{2}+4|\rho _{21}|^{2}}\right] , \beta _{\pm }=\frac{1}{2}\left[ (\rho _{33}-\rho _{44})\pm \sqrt{(\rho _{33}-\rho _{44})^{2}+4|\rho _{43}|^{2}}\right] . \end{aligned}$$

## Results and discussion

In our computations, we use typical indium arsenide (InAs) factors $m= m_{o}$, $l_{o}\approx 100 nm$, $\alpha _{r}=1.0\times 10^{-11}eV m$, and $\textit{g}=-8$^14–16, 24, 27, 29^. Also, to prepare our results we use the Wolfram Mathematica 11 software.

**Figure 1:**
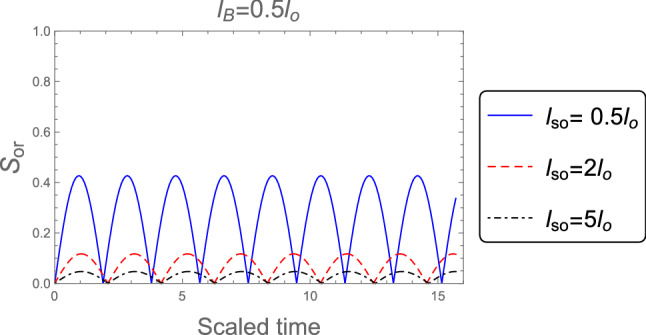
The orthogonality, $S_{or}$, when the system’s initial state in the pure state $|\Psi (0)\rangle = |\text {e}, 0\rangle$, for distinct values of SOC strength $l_{so} : l_{so}= 0.5 l_{o}, 2 l_{o}$, and $5 l_{o}$ when the robust magnetic field $l_B =0.5 l_o$ is employed.

In Fig. 1 we analyze the effect of the spin-orbit coupling $( l_{so} )$ on the orthogonality, $S_{or}$, when the system’s initial state in the pure state $|\Psi (0)\rangle = |\text {e}, 0\rangle$ with a strong magnetic field $l_B =0.5 l_o$ is employed. We observe that, the orthogonality has regular and periodic oscillations. Moreover, the orthogonality speed depends on the strength of the SOC, as the spin-orbit coupling is increased the orthogonality speed is decreased and the number of the oscillations decreased since the period increases with increasing the SOC and therefore the orthogonality speed is decreased. This indicates that when the SOC increases, the probability of transferring the information decreases.

**Figure 2:**
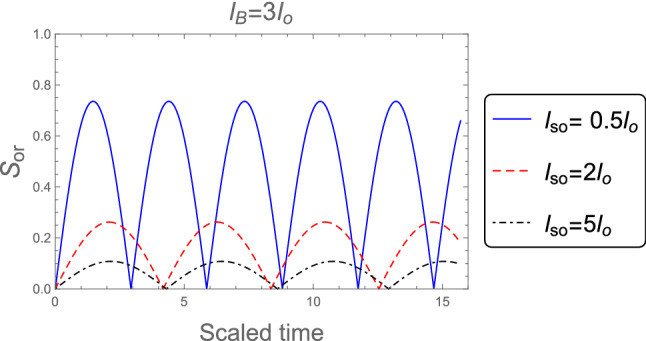
As Fig. 1, but when the weak magnetic field $l_B =3 l_o$ is applied.

**Figure 3:**
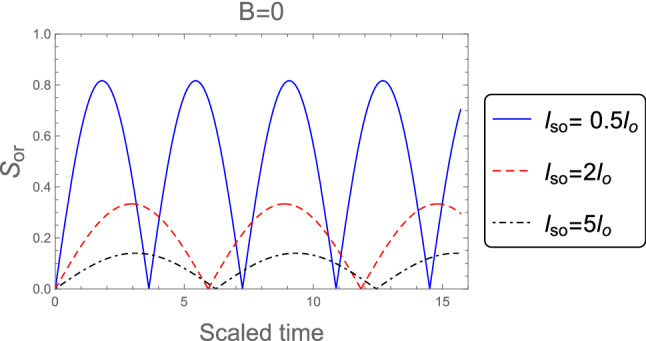
As Fig. 1, but in the absence of the magnetic field $B =0$.

Figure 2 shows the dynamics of the orthogonality when the weak magnetic field $l_B =3 l_o$ is employed, for different values of the SOC strength $l_{so} : l_{so}= 0.5 l_{o}, 2 l_{o}$, and $5 l_{o}$. It is clear that, the orthogonality number is less than in the case of strong magnetic field which indicates to the orthogonality speed is decreased in the weak magnetic field case. Also, the behavior of the orthogonality in the absence of magnetic field $B = 0$ for distinct values of the SOC strength when the system’s initial state in the pure state is described in Fig. 3. The behavior of the orthogonality speed is similar to that illustrated in Figs. 1 and 2 for strong and weak magnetic fields, although the number of oscillations and amplitude of the orthogonality speed are clearly different. When there is no magnetic field, the number of oscillations is less than when there is a strong or weak magnetic field, while the amplitudes of the orthogonality speed are larger than the case of strong and weak magnetic fields. This means that as the magnetic field becomes weak until reaches zero the orthogonality speed decrease. So, any change in the magnetic field or the intensity of the spin-orbit coupling can affect the behavior of the orthogonality speed.

**Figure 4:**
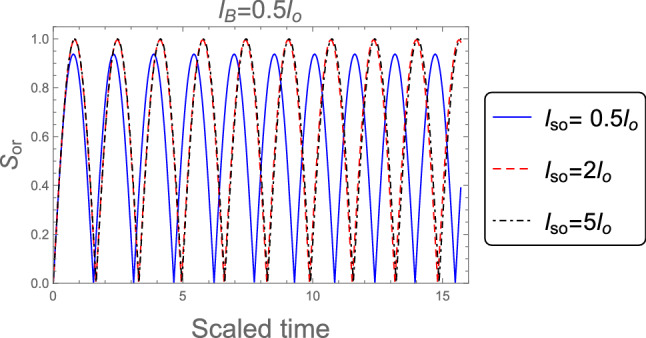
The orthogonality, $S_{or}$, when the system’s initial state in the maximum entangled state $|\Psi (0)\rangle = \frac{1}{\sqrt{2}}(|\text {e}, 0\rangle +|\text {g}, 1\rangle )$, for distinct values of SOC strength $l_{so} : l_{so}= 0.5 l_{o}, 2 l_{o}$, and $5 l_{o}$ when the robust magnetic field $l_B =0.5 l_o$ is employed.

In Fig. 4 we investigate the time evolution of the orthogonality when the system’s initial state in the maximum entangled state with $|\Psi (0)\rangle = \frac{1}{\sqrt{2}}(|\text {e}, 0\rangle +|\text {g}, 1\rangle )$, and the strong magnetic field $l_B =0.5 l_o$ is employed. In addition, The spin-orbit coupling effect on the orthogonality speed is investigated. From Fig. 4 one can see that, By increasing the intensity of the SOC the orthogonality speed decreasing gradually, and the orthogonality number is larger than in the case of pure state as obtained in Fig. 1. This means that the orthogonality speed can be controlled by the initial states of the the nanowire model.

**Figure 5:**
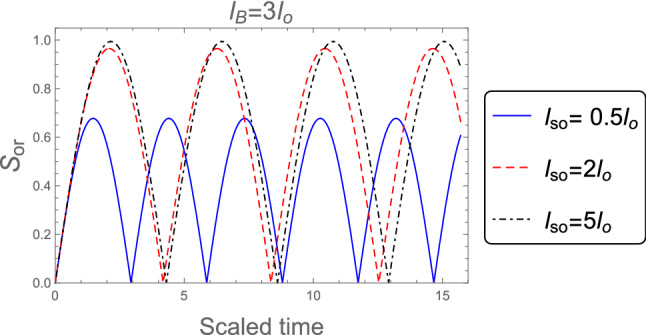
As Fig. 4, but when the weak magnetic field $l_B =3 l_o$ is applied.

**Figure 6:**
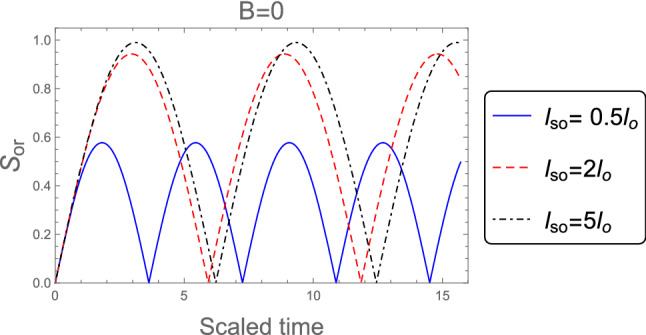
As Fig. 4, but without magnetic field $B =0$.

Figures 5 and 6 are plotted for a weak magnetic field ( $l_B =3 l_o$ ) and in the absence of magnetic field ( $B = 0$ ) with various values of the SOC strength $l_{so} : l_{so}= 0.5 l_{o}, 2 l_{o}$, and $5 l_{o}$. In comparison to the strong magnetic field case, we note the following impacts: (i) the number of oscillations for $l_B =3 l_o$ and $B = 0$ are less than that for $l_B =0.5 l_o$ and become more apparent. (ii) the amplitudes of the orthogonality for $l_B =0.5 l_o$ are larger than that for $l_B =3 l_o$ and $B = 0$. As a result, the orthogonality speed of the proposed model is mostly determined by the magnetic field values and the system’s initial states.

**Figure 7:**
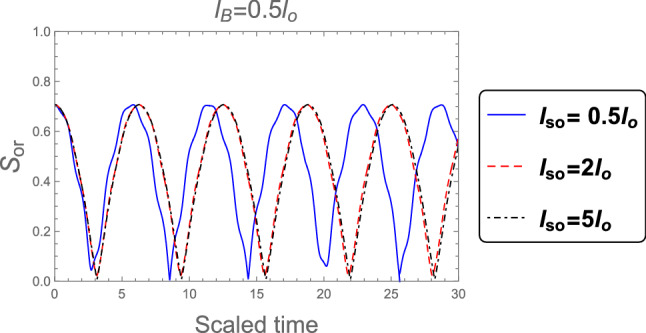
The orthogonality, $S_{or}$, when the system’s initial state in the superposition state $|\Psi (0)\rangle = \frac{1}{2}(|\text {g}, 0\rangle +|\text {g}, 1\rangle +|\text {e}, 0\rangle +|\text {e}, 1\rangle )$, for distinct values of SOC strength $l_{so} : l_{so}= 0.5 l_{o}, 2 l_{o}$, and $5 l_{o}$ when the robust magnetic field $l_B =0.5 l_o$ is employed.

Now, suppose that the system’s initial state in the superposition state $|\Psi (0)\rangle = \frac{1}{2}(|\text {g}, 0\rangle +|\text {g}, 1\rangle +|\text {e}, 0\rangle +|\text {e}, 1\rangle )$. In Fig. 7, we analyze the impacts of the SOC strength on the orthogonality speed, when the robust magnetic field $l_B =0.5 l_o$ is employed. It is clear that, the orthogonality speed has irregular oscillations on the contrary with the case of pure and maximum entangled state, but become regular with increasing the SOC. Consequently, there is a negative relationship between the SOC strength and the orthogonality speed, as the spin-orbit coupling increases, the orthogonality speed decrease since the orthogonality time increases with SOC.

**Figure 8:**
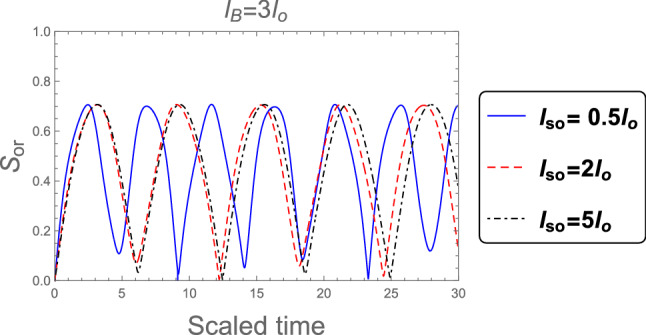
As Fig. 7, but when the weak magnetic field $l_B =3 l_o$ is applied.

**Figure 9:**
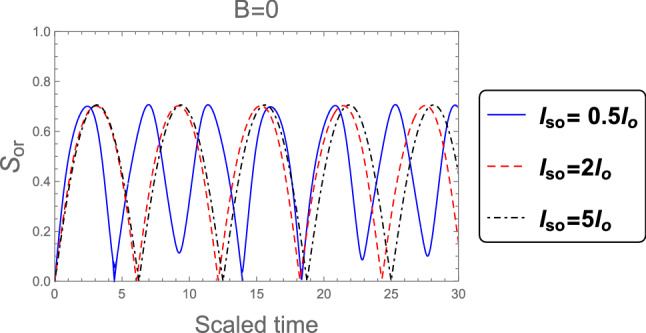
As Fig. 7, but without magnetic field $B =0$.

The dynamic of the orthogonality when the weak magnetic field ( $l_B =3 l_o$ ) and no magnetic field ( $B = 0$ ) are applied in the superposition state is shown in Figs. 8 and 9 respectively. It is seen that, the orthogonality number increases with increasing the SOC strength and Consequently the computations speed has the same behavior in the case of strong magnetic field that shown in Fig. 7 with some differences in the number of oscillations.

**Figure 10:**
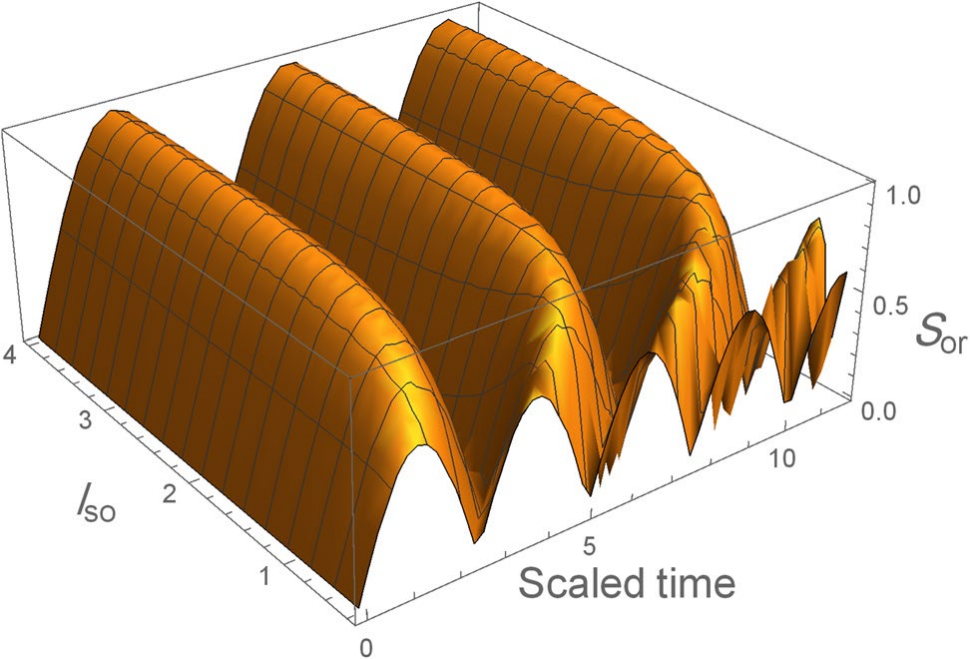
The orthogonality, $S_{or}$, as a function of spin-orbit coupling ($l_{so}$) when the system’s initial state in the maximum entangled state $|\Psi (0)\rangle = \frac{1}{\sqrt{2}}(|\text {e}, 0\rangle +|\text {g}, 1\rangle )$, when the weak magnetic field $l_B =3l_o$ is employed.

**Figure 11:**
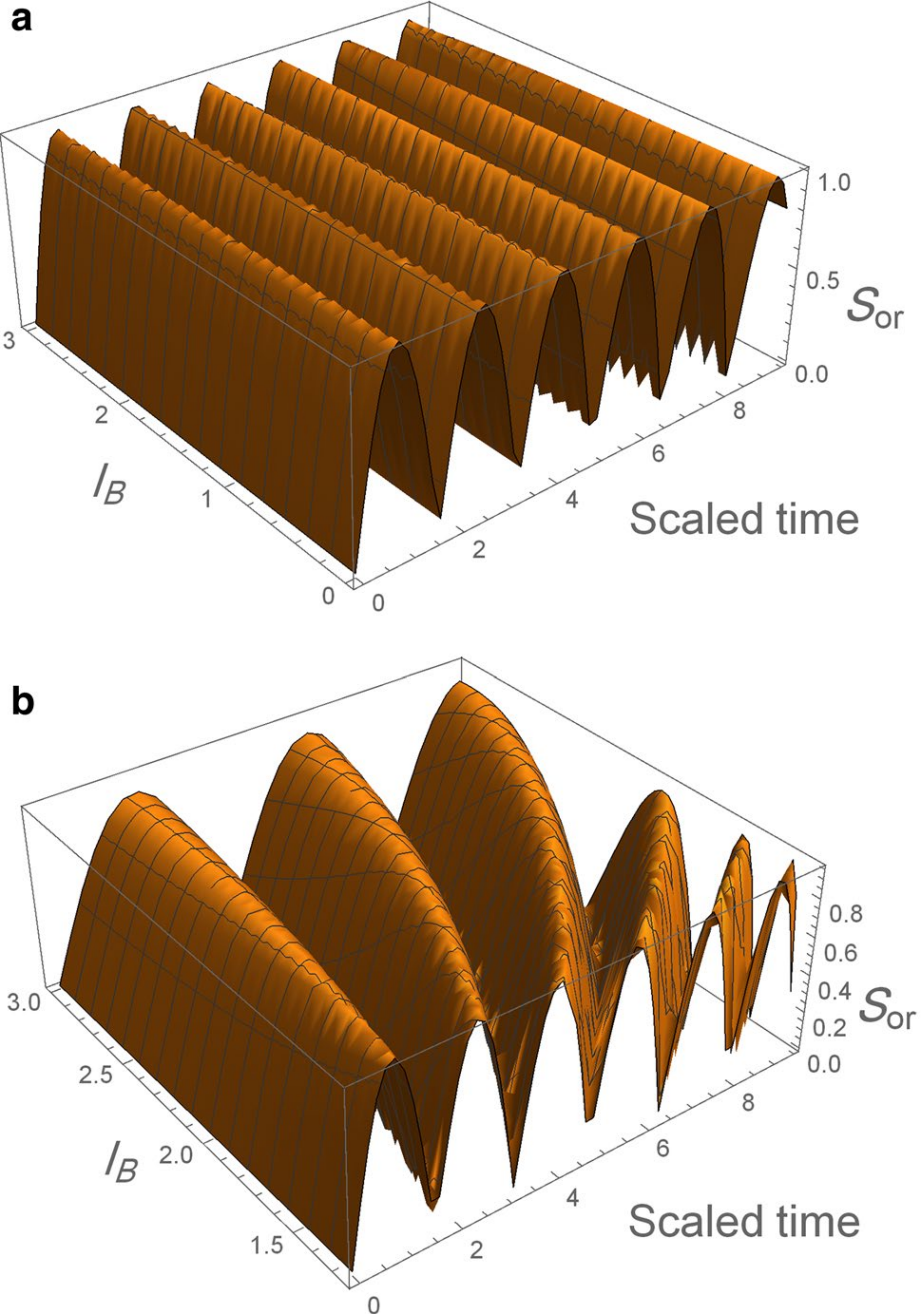
The orthogonality, $S_{or}$, as a function of magnetic field ($l_{B}$) when the system’s initial state in the maximum entangled state $|\Psi (0)\rangle = \frac{1}{\sqrt{2}}(|\text {e}, 0\rangle +|\text {g}, 1\rangle )$ and we keep $l_{so} = l_{o}$ . For strong magnetic field in (**a**) and weak magnetic field in (**b**).

Figure 10 show the orthogonality, $S_{or}$, as a function of the spin-orbit coupling ($l_{so}$) with the system’s initial state in the maximum entangled state $|\Psi (0)\rangle = \frac{1}{\sqrt{2}}(|\text {e}, 0\rangle +|\text {g}, 1\rangle )$ when the weak magnetic field $l_B =3l_o$ is employed, we observe that the period and the amplitude of the orthogonality increases with increasing the SOC, which implies to the orthogonality speed decrease with the SOC that agree with the results in all figures above. While Fig. 11 show the orthogonality as a function of the magnetic field in the maximum entangled state and we keep $l_{so} =l_o$, it is clear that the orthogonality number in the case weak magnetic field is less than the strong magnetic field case which indicates to the orthogonality speed is decreased when the magnetic field become weak.

We may deduce that our results agree with the results in Ref.^6^ where the orthogonality speed for a single qubit system interacting with a quantized field has been studied, and the effect of the coupling constant on the speed of orthogonality is investigated, as one increases the coupling constant the speed of orthogonality decreases which is the same role for the spin-orbit coupling in our result where the orthogonality speed decrease with increasing the spin-orbit coupling (SOC).

A summary of the results above, the computations speed of a nanowire system is sensitively affected not only by the intensity of the magnetic field and the spin-orbit coupling, but also by the system’s initial states.

## Conclusion

The quantum computational speed of nanowire system with different types of magnetic fields when the initial states are prepared in various forms has been examined via the orthogonality speed. The influence of the magnetic field, the spin-orbit coupling, and the system’s initial state on the computational speed have been discussed. Our results demonstrate that, the intensity of the SOC is critical in decreasing the orthogonality numbers, as the SOC strength increases, the orthogonality numbers decrease. Moreover, the speed of orthogonality can be manipulated by the type of magnetic field, where the shortest orthogonality time occurs when the strong magnetic field is employed, while the longest duration occurs in the absence of a magnetic field. Finally, the system’s initial states have a significant influence on the orthogonality speed, where the orthogonality speed in a maximum entangled state is faster than in the pure or superposition states. This means that, the magnetic field, the spin-orbit coupling, and the system’s initial state all have a significant effect on the quantum computational speed of the nanowire system. As a result, the nanowire SOC and magnetic fields will slow down the computational speed.
